# Saturated Fatty Acids Modulate Cell Response to DNA Damage: Implication for Their Role in Tumorigenesis

**DOI:** 10.1371/journal.pone.0002329

**Published:** 2008-06-04

**Authors:** Li Zeng, Guang-Zhi Wu, Kim Jee Goh, Yew Mun Lee, Chuo Chung Ng, Ang Ben You, Jianhe Wang, Deyong Jia, Aijun Hao, Qiang Yu, Baojie Li

**Affiliations:** 1 Cancer and Developmental Biology Division, Institute of Molecular and Cell Biology, Singapore, Republic of Singapore; 2 China-Japan Union Hospital, Jilin University, Changchun, Jilin, People's Republic of China; 3 The Key Laboratory of Experimental Teratology, Ministry of Education, Faculty of Medicine, Shandong University, Jinan, Shandong, People's Republic of China; 4 Laboratory of Molecular Pharmacology, Genome Institute of Singapore, Singapore, Republic of Singapore; University of Hong Kong, China

## Abstract

DNA damage triggers a network of signaling events that leads to cell cycle arrest or apoptosis. This DNA damage response acts as a mechanism to prevent cancer development. It has been reported that fatty acids (FAs) synthesis is increased in many human tumors while inhibition of fatty acid synthase (FASN) could suppress tumor growth. Here we report that saturated fatty acids (SFAs) play a negative role in DNA damage response. Palmitic acid, as well as stearic acid and myristic acid, compromised the induction of p21 and Bax expression in response to double stranded breaks and ssDNA, while inhibition or knockdown of FASN enhanced these cellular events. SFAs appeared to regulate p21 and Bax expression via Atr-p53 dependent and independent pathways. These effects were only observed in primary mouse embryonic fibroblasts and osteoblasts, but not in immortalized murine NIH3T3, or transformed HCT116 and MCF-7 cell lines. Accordingly, SFAs showed some positive effects on proliferation of MEFs in response to DNA damage. These results suggest that SFAs, by negatively regulating the DNA damage response pathway, might promote cell transformation, and that increased synthesis of SFAs in precancer/cancer cells might contribute to tumor progression and drug resistance.

## Introduction

Numerous studies have implicated that fatty acids, fat diet, and obesity play a role in cancer development [1]–[3]. Fatty acids are the building blocks of fat and exist either in free forms or components of triacylglycerol, phospholipids, and cholesterol. In serum, the concentration of free fatty acids is >500 µM under normal conditions and >1200 µM under fasting, with palmitic acid accounting for 28% [4], [5]. They can be obtained from the diet fat or synthesized in the cells, especially in lipogenic tissues such as liver, adipose, and lactating breast. Fatty acids are synthesized by FASN using malonyl-CoA and acetyl-CoA as substrates. For people with a balanced diet, *de novo* fatty acid synthesis is insignificant and FASN protein level is very low in lipogenic as well as other tissues. FAs play important roles in energy storage, membrane structure, protein acylation, signal transduction, and regulation of gene transcription [6].

However, cancer cells, especially of the breast, prostate, colon, ovary, endometrium, and thyroid origin, express very high levels of FASN and this up-regulation is under the control of aberrant MAPK and PI-3K-Akt signaling [2], [7]–[9]. FASN is also expressed in early stages of tumor development or pre-cancer lesions such as colonic adenoma, dysplastic squamous epithelium, and carcinoma of the tongue, although this up-regulation is more pronounced in the late stages of tumors. Moreover, FASN can be detected in the serum of these patients and this can be used as a diagnostic marker. *De novo* synthesized fatty acids account for more than 90% of the triacylglycerol in tumor cells [10]. Exacerbated FAs metabolism is believed to play an important role in cancer pathogenesis by conferring proliferating advantage [1].

FASN is now becoming a drug target for cancer therapy. It has been found that cerulenin, a natural fungal inhibitor of FASN, specifically targets and suppresses tumor cell growth, with little effect on the surrounding normal tissues [11]. A small compound, called C75, has a similar efficacy on FAs synthesis and anti-tumor activity [12]. These compounds inhibit cell cycle progression and causes apoptosis [13]. These effects seem to be mediated by FAs synthesis. However, how inhibition of FASN suppresses tumor growth remains unclear. Another question is the roles of increased synthesis of FAs in tumorigenesis.

DNA damage is generated by exogenous agents such as ionizing radiation (IR), ultraviolet (UV) light exposure, genotoxic compounds including chemotherapeutic drugs such as adriamycin, and endogenous factors such as reactive oxygen species which are generated by mitochondria in the process of β-oxidation. Depending on the types and the severity of DNA lesions, cells respond to DNA damage by undergoing cell cycle arrest or apoptosis when the damage is beyond repair [14].

DNA damage activates multiple signaling cascades. At the center of the signaling network are phosphoinositide-3-kinase-like kinases (PIKKs) that include DNA-PKcs, Atm, and Atr, all of which are exclusive serine/threonine kinases [15]. Atm responds mainly to double stranded breaks (DSB), while Atr is activated by single stranded DNA (ssDNA), stalled DNA replication and UV-induced damage. DSBs alter the chromatin structures and induce rapid intermolecular phosphorylation of Atm on Ser1981, leading to dissociation of the previously inert dimer complex and activation of Atm. Activated Atm initiates cell signaling events to induce cell cycle arrest or apoptosis through phosphorylation of p53 at Ser15 and up-regulation of p21 or Bax and Puma [16]. Later, DSBs can be converted to ssDNA during repair, where Atr is recruited and activated [17]. Activated Atr can phosphorylate p53 and Chk1 to regulate cell cycle and apoptosis.

Many of the proteins involved in DNA damage response are found to promote cancer development when mutated [18]. Furthermore, it was recently reported that in many cell types, the conversion from pre-cancer to cancer is accompanied by activation of the DNA damage response, which ceases to exist once converted to cancer cells [19], [20]. The function for this activation is to inhibit cell proliferation or to induce apoptosis. As a result, cells with mutations in proteins involved in DNA damage response are selected and become cancerous. Thus, DNA damage response acts as a protective mechanism against cancer development [16]. Since FASN expression is up-regulated and FAs levels are increased in precancer as well as cancer cells, we studied the role of SFAs in DNA damage response by checking p53 accumulation, p21 and Bax induction, and cell growth. We found that the presence of SFAs compromised cell response to DNA damage in primary cells but not in immortalized or transformed cells, at a step upstream of p53 and Chk1, likely Atr. Moreover, the observation that SFAs have a more dramatic effect on p21 and Bax induction than on p53 phosphorylation and stabilization in response to DNA damage and that SFAs could regulate p21 and Bax expression in the absence of genotoxic stress suggests that SFAs also control p21 and Bax expression through other pathway(s) in addition to Atr-p53. These results support the concept that increased FAs synthesis might promote tumorigenesis by downplaying the DNA damage response pathway.

## Results

### A negative role for SFAs in DNA damage response in primary MEFs

**Figure 1:**
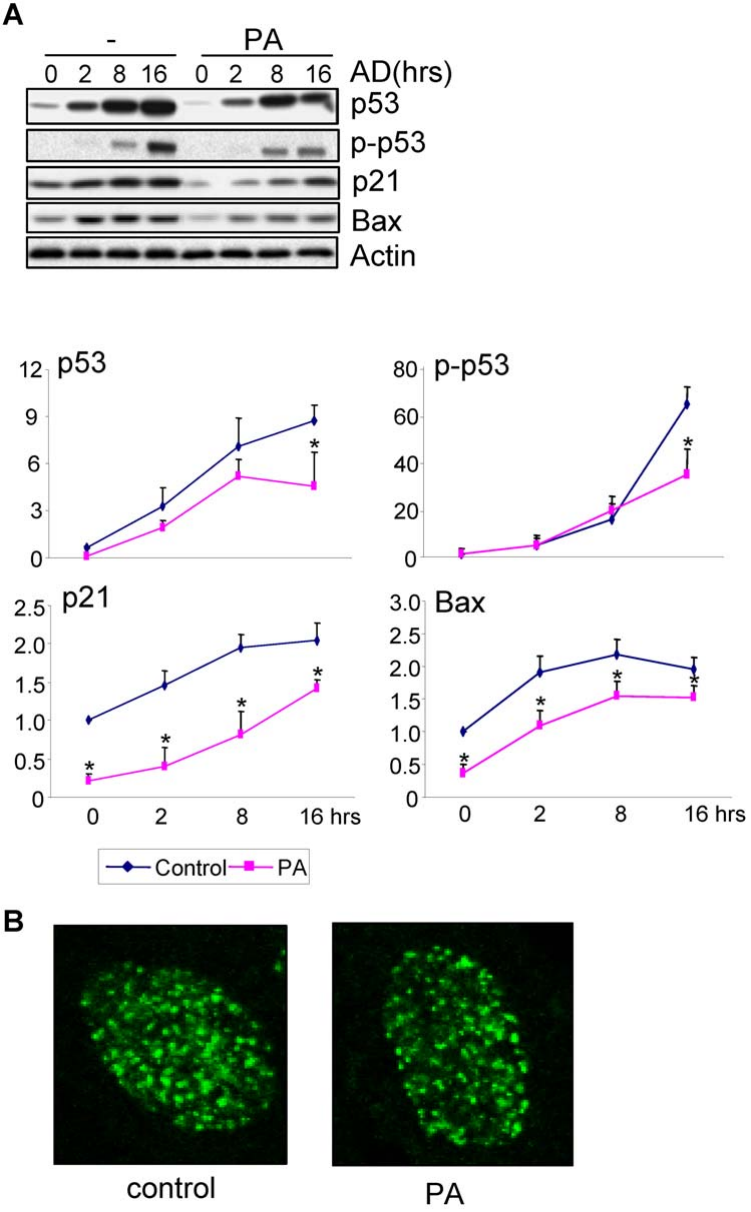
SFA inhibited adriamycin-induced p53 activation and induction of p21 and Bax in MEFs. A. MEFs were pretreated with 100 µM of palmitic acid (PA) for 16 hrs and then stressed with adriamycin for different periods of time. Cells were collected and the protein levels of p53, p21, Bax, and β-Actin and the level of phospho-p53 (Ser15) were determined by Western blot analysis. Bottom panels: quantitation data. The values of signals at different time points were normalized to that of time 0, which were set at 1.0 without SFA treatment. Asterisks mark samples significantly different from the control group at the same time point with P<0.05. B. Palmitic acid showed no effect on adriamycin-induced nuclear foci assembly of H2AX. MEFs were pretreated with palmitic acid overnight, then with adriamycin for 8 hrs, and immuno-stained for endogenous H2AX. Counting the number of foci revealed no significant difference between PA-treated and untreated cells.

Since DNA damage response plays an important role in preventing tumorigenesis and FAs synthesis is greatly enhanced in pre-cancer/cancer cells, we attempt to test whether SFAs affect DNA damage response. We first tested palmitic acid, the most abundant SFA in serum, the end-product of *de novo* fatty acid synthesis, and a substrate for lipid synthesis and protein palmitoylation. Primary mouse embryonic fibroblasts (MEFs) were used since they show good response to DNA damage induced by adriamycin, a chemotherapeutic drug that has been proved to cause both DSBs and ssDNA. Treatment of MEFs with different doses of palmitic acid (50, 100, 200, 300 µM) revealed that palmitic acid was toxic to the cells at concentrations over 200 µM (data not shown). Therefore, 100 µM of palmitic acid was used throughout this study. We also found that palmitic acid itself slightly down-regulated the basal levels of p21 and Bax in MEFs (Fig. 1A), suggesting that palmitic acid can also regulate the expression of these molecules via pathways that are not activated by DNA damage. Moreover, palmitic acid significantly inhibited adriamycin-induced up-regulation of p21, Bax, and p53, as well as phosphorylation of p53 on Ser15 (Fig. 1A). However, we found that palmitic acid showed no effect on adriamycin-induced nuclear foci formation of H2AX (Fig. 1B), an indication of the extent of DNA damage. These results suggest that palmitic acid acts at a step upstream of p53 but downstream of foci formation. Furthermore, we found that palmitic acid showed a more dramatic effect on p21 and Bax induction than on p53 phosphorylation and stabilization (Fig. 1A), confirming that palmitic acid could also regulate p21 and Bax induction in a p53 phosphorylation/stabilization independent manner.

In order to investigate whether this effect is palmitic acid specific, we then tested other saturated fatty acid stearic acid (C18) and myristic acid (C14), both of which are present in the serum with stearic acid accounting for close to 13 % of the total FAs [5]. We found that pretreating MEFs with either stearic acid or myristic acid, at 100 µM, also compromised adriamycin-induced p21 and Bax induction, p53 phosphorylation, and p53 accumulation (Supplemental Fig. S1). These results suggest that SFAs play a rather general role in cell response to DNA damage.

**Figure 2:**
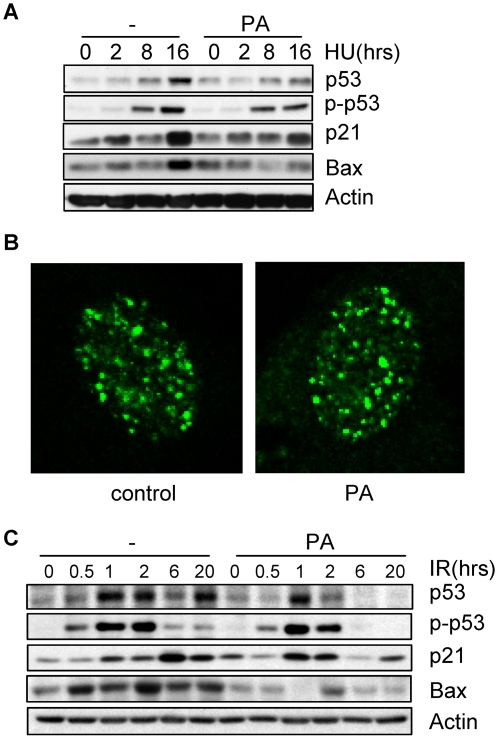
Hydroxyurea and ionizing radiation-induced DNA damage response was also inhibited by palmitic acid in MEFs. A. MEFs were pretreated with 100 µM of palmitic acid (PA) for 16 hrs and then stressed with 5 mM hydroxyurea (HU) for different periods of time. Cells were collected and the protein levels of p53, p21, Bax, p-p53, and β-Actin were determined by Western blot analysis. B. Palmitic acid showed no effect on hydroxyurea-induced nuclear foci assembly of H2AX. MEFs were pretreated with palmitic acid overnight, then with hydroxyurea for 8 hrs, and immuno-stained for endogenous H2AX. Counting the number of foci revealed no significant difference between PA-treated and untreated cells. C. Similar experiment was carried out except that DNA damage was induced by 10 Gy IR.

Another genotoxic stress reagent hydroxyurea (HU), which generates ssDNA and activates Atr, was used to confirm this finding. As expected, HU could induce p21 expression and p53 accumulation in primary MEFs (Fig. 2A). Pretreatment with palmitic acid impeded p53 and p21 up-regulation, especially at late time point, without affecting hydroxyurea induced nuclear foci formation (Fig. 2A and 2B, and Supplemental Fig. S2A). These results support a role for palmitic acid in cell response to ssDNA. IR, which initially generates DSB and rapidly activates Atm, and later ssDNA in the subsequent repair process and activates Atr [17], was also examined. In general, we found that palmitic acid pretreatment inhibited p53 phosphorylation and up-regulation of p53, p21, and Bax, notably at later time points, without affecting nucleus foci formation (Fig. 2C, Supplemental Fig. S2B, and data not shown). These results suggest that SFAs might have a more prominent effect on Atr. Surprisingly, we found that palmitic acid treatment also led to a quicker induction of p21 in response to IR (compare the 1, 2, 6 hr treatment of adriamycin in Fig. 2C). The discrepancy between p53 phosphorylation/stabilization and p21 induction confirms that palmitic acid can regulate p21 expression in p53 independent manner.

**Figure 3:**
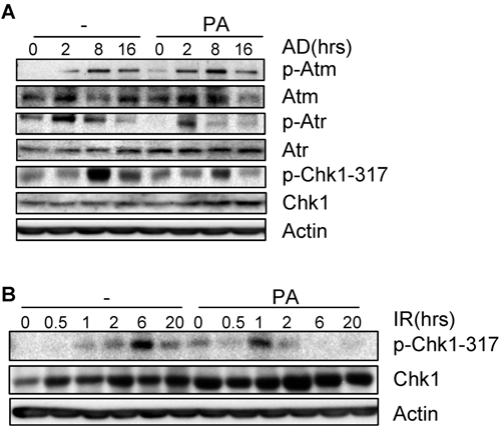
SFA inhibited phosphorylation of Atr and Chk1 in MEFs. A. MEFs were pretreated with 100 µM of PA for 16 hrs and then stressed with adriamycin for different periods of time. Cells were collected and the protein levels of Atm, phospho-Atm, Atr, phospho-Atr, Chk1, and phospho-Chk1 were determined by Western blot analysis. B. Similar experiment was carried out except that DNA damage was induced by 10 Gy IR.

In support of this notion, we also found that adriamycin-induced Atm activation was not significantly altered by pretreatment with palmitic acid in MEFs (Fig. 3A and Supplemental Fig. S3A). Since DNA damage-induced foci and the activation of Atm are indications of the extent of DNA damage, we believe that the presence of SFAs do not have an effect on the amounts of damaged DNA generated by adriamycin treatment. On the contrary, phosphorylation of Atr was reduced in the presence of palmitic acid at the basal level or in response to adriamycin treatment. In accordance, Chk1 phosphorylation at Ser317, a substrate specific for Atr, was also reduced (Fig. 3A and Supplemental Fig. S3A). This is consistent with the observation that HU-induced DNA damage response was negatively regulated by SFAs (Fig. 2A) and that IR-induced Chk1 phosphorylation was also diminished in palmitic acid pretreated cells (Fig. 3B and Supplemental Fig. S3B). Moreover, inhibition of Atm and Atr with caffeine blocked adriamycin induced up-regulation of p21, Bax, p53, and p53 phosphorylation (Supplemental Fig. S4). Treatment with both caffeine and palmitic acid gave rise to similar results as caffeine alone (Supplemental Fig. S4). These results suggest that Atm and Atr are essential for the DNA damage response under our experimental settings and that SFAs' effect on DNA damage response is likely to be mediated by Atm and Atr, especially Atr (based on Fig. 3). Further investigation is needed to understand the mechanisms by which palmitic acid regulates the activation of Atr.

### Inhibition of fatty acid synthase enhanced p21 induction and p53 accumulation

**Figure 4:**
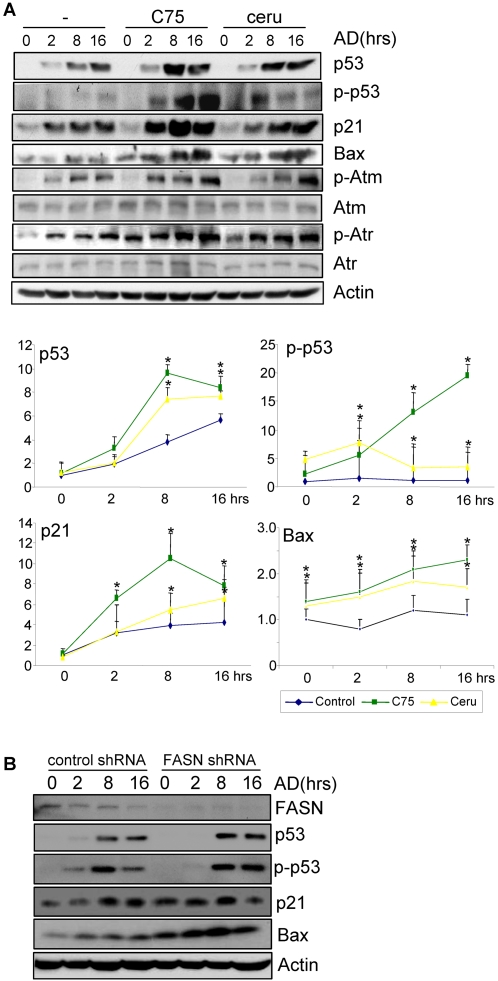
Inhibition of fatty acid synthesis with Cerulenin and C75 enhanced DNA damage response in MEFs. A. MEFs were pretreated with 1 µg /ml of Cerulenin (Ceru) or C75 for 16 hrs and then stressed with adriamycin for different periods of time. Cells were collected and the protein levels of p53, p21, Bax, and β-Actin and the level of phospho-p53 (Ser15) were determined by Western blot analysis. Bottom panels: quantitation data (fold induction). B. Knockdown of FASN with shRNA showed increased p53 phosphorylation and induction of p21 and Bax in response to adriamycin. Asterisks mark samples significantly different from the control group at the same time point with P<0.05.

To further substantiate the conclusion that SFAs have an influence on DNA damage response, we inhibited FA synthesis by adding FASN inhibitors and then checked DNA damage response. MEFs were pretreated with 1 µg/ml C75 or cerulenin, which have been shown to inhibit fatty acid synthesis in human or mouse fibroblasts [11], [23]–[25], and then challenged with adriamycin. It was found that C75 or cerulenin markedly enhanced p-Atr, p21/Bax induction, p53 phosphorylation, and p53 accumulation (Fig. 4A and Fig. S5A), with C75 exhibiting a stronger effect than cerulenin. Such a difference between these two inhibitors has been previously reported [26], [27]. Moreover, these findings were also confirmed by the FASN knockdown experiments (Fig. 4B and Fig. S5B). FASN was knocked down in MEFs with pooled shRNA. When treated with adriamycin, these cells showed enhanced induction of p21, Bax and p53, and phosphorylation of p53 (Fig. 4B). These results suggest that lowering SFA had a positive effect on this response, which further supports that SFAs play a negative role in DNA damage response. It is predictable that up-regulated expression of FASN and increased levels of FAs observed in different cancer types would compromise cell response to DNA damage, thus promoting tumorigenesis. It has been previously reported that C75 could trigger DNA damage response by activating p53 and inducing p21 expression [28]. However, we found that the levels of p53 and p21 did not markedly change in the presence of C75 or cerulenin in MEFs (Fig. 4A).

### SFAs also impaired cell response to DNA damage in primary osteoblasts

**Figure 5:**
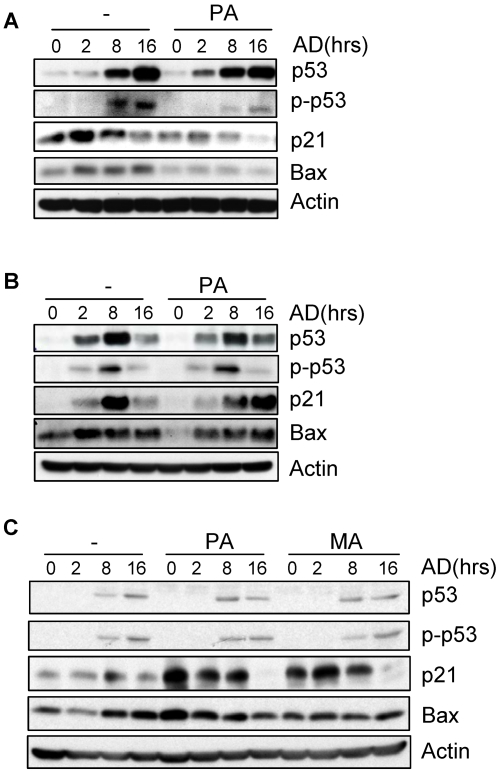
Murine primary osteoblasts, NIH3T3, and MCF-7 showed different responses to the effects of SFA. Primary calvarial osteoblasts (A), NIH3T3 (B), and MCF-7 (C) cells were pretreated with 100 µM of PA or MA for 16 hrs and then stressed with adriamycin for different periods of time. Cells were collected and the protein levels of p53, p21, Bax, and β-Actin and the level of phospho-p53 (Ser15) were determined by Western blot analysis.

In order to exclude a cell type specific effect, we also tested primary osteoblasts that were freshly isolated from the calvaria of new born pups or fetuses. These cells are of bone marrow mesenchymal stem cell origin. It was found that these cells responded to adriamycin treatment to a similar extent as primary MEFs, justified by increased expression of p21 and accumulation of p53. Similarly, pretreatment of these cells with palmitic acid compromised p21 and Bax induction and p53 phosphorylation (Fig. 5A and Supplemental Fig. S6A), suggesting that the function for SFAs in DNA damage response is also applicable to primary osteoblasts. However, palmitic acid treatment showed an insignificant effect on the protein levels of p53 in response to adriamycin (Fig. 5A). This could be due to a cell type specific effect on p53 stabilization. Stabilization of p53 can also be regulated by other proteins such as Atf3, which is induced by DNA damage via MAPK pathways [29].

### SFAs showed no inhibitory effect on DNA damage response in immortalized NIH3T3 and cancer cell lines MCF-7 and HCT116

We also expanded our study to include immortalized and tumor cell lines. NIH3T3 cells appear to be a good match for primary MEFs as they share the same origin. Upon DNA damage, NIH3T3 showed response to DNA damage through induction of p21 and accumulation of p53, even though phosphorylation of p53 was modest (Fig. 5B and Fig. S6B). Unlike primary MEFs, pretreating the cells with palmitic acid showed little effect on the protein levels of Bax, p21 and p53, and p53 phosphorylation in response to adriamycin. Moreover, in this cell line, SFAs hardly showed any effects on hydroxyurea-induced DNA damage response either (data not shown). We tried to use other immortalized MEFs to confirm this finding. Unfortunately, we were unable to get any immortalized MEFs that do not have mutations in p53 (data not shown). We then tried human breast cancer cell line MCF-7 that is shown to express high levels of FASN [9]. Palmitic acid or myristic acid treatment showed little effect on adriamycin induced p53 phosphorylation or p53 up-regulation. Yet we observed an increase in the basal levels of p21 and Bax in the presence of palmitic acid or myristic acid. Adriamycin treatment did not further induce the expression of p21 and Bax (Fig. 5C and Fig. S6C). This confirms that SFAs might regulate p21 and Bax in the absence of DNA damage.

**Figure 6:**
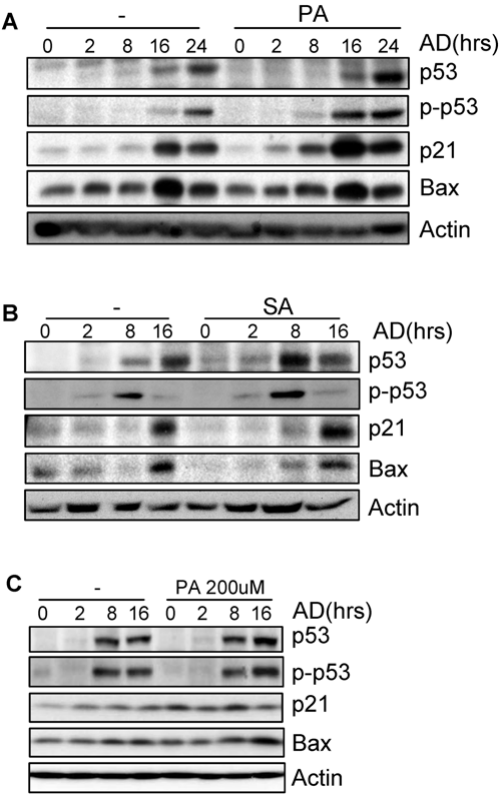
SFA did not repress the DNA damage response in HCT116 cells. A. HCT116 cells were pretreated with 100 µM of PA for 16 hrs and then stressed with adriamycin for different periods of time. Cells were collected and the protein levels of p53, p21, Bax, and β-Actin and the level of phospho-p53 (Ser15) were determined by Western blot analysis. B. The effect of SA in HCT116 cells. C. Palmitic acid, even at 200 µM, did not repress adriamycin-induced p-53 phosphorylation or induction of p21 and Bax.

We further examined this on human colon cancer cell line HCT116 that expresses wild type p53. This cell line has been shown to highly express fatty acid synthase and likely to contain high levels of FAs [9]. It also responded well to adriamycin in terms of p53 accumulation and p21 induction. However, unlike primary cells, pretreatment of these cells with palmitic acid or stearic acid did not inhibit the induction of p21 or Bax, instead, a slightly enhanced induction was observed for p21 (Figs. 6A, 6B, S7A and S7B). Moreover, palmitic acid, even at 200 µM, a dosage showing toxic effect in HCT116 cells, failed to repress adriamycin-induced up-regulation of p53, p21, and Bax (Fig. 6C and Fig. S7C). These results confirmed that SFAs differentially regulate DNA damage response in primary and transformed cells. We then tried FASN inhibitor C75 in HCT116 cells. It was found that C75, at 1 or 5 µg/ml, enhanced the up-regulation of p21, Bax, and p53, without affecting p53 phosphorylation on Ser15 (Supplementary data Fig. S8). This is consistent with a previous study showing that pharmacological inhibition of FASN induced p53 and p21 up-regulation in RKO colon carcinoma cells [28]. Therefore, although HCT116 cells behaved differently from primary MEFs in the presence of SFAs, they seem to respond to FASN in a similar way. This discrepancy between primary cells and immortalized/transformed cells could be due to the high levels of *de novo* synthesized FAs.

### SFAs promoted cell proliferation in the presence of adriamycin

**Figure 7:**
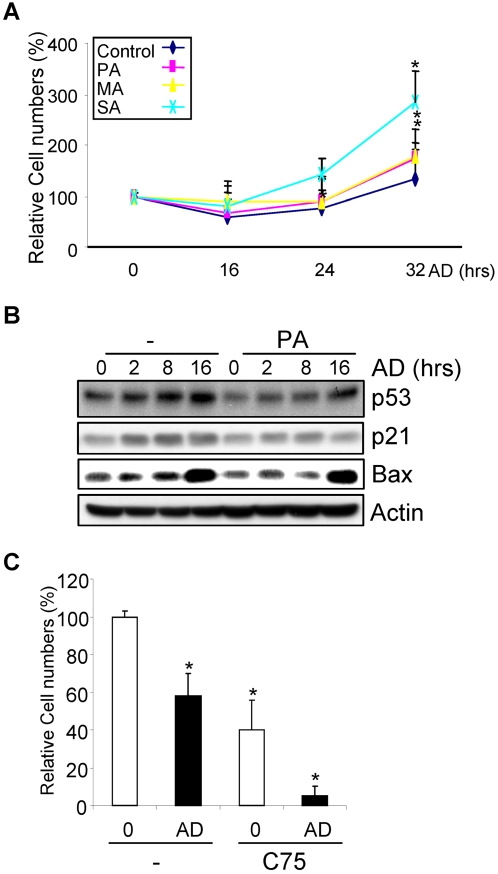
SFA showed an inhibitory effect on adriamycin-induced cell death. A. MEFs were pretreated with 100 µM of PA, MA, or SA for 16 hrs and then stressed with 0.1 µM adriamycin for different periods of time. The number of viable cells was determined by WST-1 assay. The experiments were repeated three times. B. Adriamycin, at 0.1 µM, was able to activate DNA damage response pathway. C. Adriamycin and C75 showed a synergistic effect in inducing cell death in MEFs. Asterisks mark samples significantly different from the control group at the same time point with P<0.05.

DNA damage is known to induce cell cycle arrest and/or apoptosis. We found that palmitic acid had little effect on the distribution of G1, S, and G2 phases, in the presence or absence of adriamycin, suggesting that its effect on cell cycle checkpoints is minimal (data not shown). On the other hand, several studies have reported that SFAs, especially palmitic acid, could regulate apoptosis. In some cases such as pancreatic cells, palmitic acid induces cell death through mtDNA damage and genomic DNA damage [30], [31], probably due to the reactive oxygen species generated during beta-oxidation of acetyl-CoA. However, we did not observe a DNA damage response upon palmitic acid treatment (Fig. 1). Other reports also state that palmitic acid induces apoptosis by down-regulating cardiolipin, a phospholipid that helps the insertion and retention of cytochrome C into the mitochondria membrane. In contrast, it has also been reported that inhibition of FASN reduced both the level of palmitic acid and apoptosis, and exogenously added palmitic acid helped to protect the cells [32]. To test whether SFAs have an effect on adriamycin-induced cell death, MEFs were pretreated with 100 µM SFAs for 16 hrs and then challenged with 0.1 µM adriamycin for different periods of time. Cell proliferation/survival rates were measured with WST-1 assay. Under this setting, SFAs pretreatment could only slightly inhibit adriamycin-induced cell death (Fig. 7A). Interestingly, we found that cells could recover after prolonged treatment, and that cells pretreated with SFAs appeared to proliferate at an increased rate compared to the untreated cells (Fig. 7A). Adriamycin, even at 0.1 µM, was able to induce the expression of p21and Bax, and the stabilization of p53, although Ser15 phosphorylation was hardly detectable (Fig. 7B and data not shown). When 0.5 µM of adriamycin was applied to the cells, the stimulatory effects of SFAs on proliferation were less obvious (Supplementary data Fig. S9), likely due to the toxic effect of adriamycin. Moreover, C75, as well as cerulenin, induced cell death in MEFs. A combination of C75 with 0.1 µM of adriamycin resulted in increased cell death rates (Fig. 7C and data not shown). This finding is consistent with previous findings that FASN inhibitor could induce cell death and that these cells could be rescued by addition of FAs [33], [34].

## Discussion

Our studies indicate that SFAs could compromise DNA damage-induced cell response in primary cells but not in immortalized cells. Pretreatment with palmitic acid, myristic acid, or stearic acid leads to reduced accumulation of p53, reduced induction of p21 and Bax, diminished activation of Chk1, and improved cell proliferation. Moreover, inhibition of FAs synthesis, with pharmacological inhibitors or knock-down of FASN, enhanced the induction of p21 and Bax and accumulation of p53. Based upon the fact that the levels of FASN expression and FA synthesis are dramatically increased in the pre-cancer stages, and that DNA damage response occur during the transition from precancerous to cancer, we propose that the increased levels of FAs might modulate cell response to DNA damage, leading to defects in cell cycle checkpoints and apoptosis. Compromised DNA damage will likely result in accumulation of DNA lesions and eventually facilitate tumorigenesis. This concept is also supported by the observation that overexpression of FASN is correlated with high degree of microsatellite instability in colorectal cancer independent of CpG island methylator phenotypes [35]. The actions of SFAs in DNA damage response might also be one of the mechanisms underlying the association between high fat diet/obesity and tumorigenesis [36]. Cells with active fatty acids metabolism might have defect in cell cycle arrest or apoptosis in response to genotoxic stress. The predominant cancers associated with obesity are breast, prostate, endometrium, colon, and gallbladder cancers. Most of these cancer types express very high levels of FASN [37]. Noteworthy is that the correlation between high fat diet and increased risk for tumorigenesis is still controversial except in ovarian cancer [38].

DNA damage response is controlled by signaling pathways started from the DNA damage-induced foci, which are believed to be DNA repair centers. Many proteins were accumulated at the foci including the signaling molecules Atm and Atr, which phosphorylate p53, Mdm2, Chk1/2 and other molecules to regulate cell cycle control and apoptosis. Since SFAs interfered with p53 phosphorylation, p53 accumulation, induction of p53 target genes such as p21 and Bax, and phosphorylation of Chk1, we conclude that SFAs act at a rather early step of the signaling cascade. Since foci formation is not affected, SFA is likely to act at a step affecting Atm/Atr activation, or adaptor proteins that recruit effectors such as p53 and Chk1/2 to the foci, or both. We found that SFAs had a more profound effect on Atr phosphorylation and activation of Chk1, implying that SFAs might mainly regulate the activation of Atr and/or the phosphorylation of Atr targets. But how do SFAs execute their function on Atr? In view of this, there are a few possibilities. Firstly, SFAs might alter the synthesis of phospholipids, which are known to regulate signaling events. A recent study showed that a shift of phosphatidylcholine chains from polysaturated fatty acids to polyunsaturated fatty acids activates p53 Ser15 phosphorylation through Atr [39]. Secondly, as shown for transcription factors PPAR and LXR, SFAs and their metabolic intermediates might interact directly with signaling molecules. Thirdly, SFAs especially palmitic acid, as well as their metabolic intermediate acetyl-CoA, can be directly used as substrates to modify proteins, in the forms of palmitoylation and acylation. Protein palmitoylation has been shown to affect enzymatic activity, protein trafficking, or protein stabilization [40], [41]. In addition, it appears that in response to genotoxic stress, SFAs have a more dramatic effect on the induction of p21 and Bax than on p53 phosphorylation and stabilization. It was also found that in some cell types, SFA could regulate the expression of p21 and Bax in the absence of genotoxic stress. Therefore, SFAs regulate the Atr-p53-p21/Bax pathway at more than one step. Further investigation is needed to understand the mechanisms by which SFAs regulate cell response to DNA damage.

One intriguing finding is that while primary cells' response to DNA damage is repressed by SFAs, immortalized/transformed cells show resistance to this effect. One possible explanation is that immortalized/transformed cells have acquired some changes in the gene expression patterns, in order to adapt to the long term presence of elevated levels of FASN and fatty acids. So they will not respond to further increases in SFAs. The second possibility is that the immortalized/transformed cells might have acquired mutations in the proteins that link fatty acids to the DNA damage response and repair pathways, e.g., proteins involved in DNA damage response that can bind or be modified by SFAs. The observation that both primary and transformed cells are responsive to FASN inhibition in genotoxic stress favors the first hypothesis as genetic changes are usually irreversible. Another interesting finding is that SFAs only modulate cell survival and proliferation in response to low dose of adriamycin, which might reflect the *in vivo* condition, where the nutrients SFAs modulate cell response to modest DNA damage generated by endogenous factors such as ROS. These results also suggest that SFAs may play a minimal role in DNA damage caused by high dose of genotoxic agents.

Inhibitors of FASN have been found to induce apoptosis in cancer cells that have high levels of FASN. These include breast, prostate, colon, and lung cancers. Based upon our findings that an increase in SFA levels compromises DNA damage response while inhibition of FAs synthesis enhances these cellular events, lowering the cellular level of FAs might reduce the risks of cancer development. In addition, lowering the cellular level of FAs might also improve the efficacy of radiotherapy and chemotherapy with genotoxic drugs. Some chemotherapeutic drugs such as cyclophosphamide, busulfan, cisplatin, and mitomycin cause interstrand and/or intrastrand crosslinks, while some, e.g., irinotecan and dactinomycin, affect DNA unwinding and therefore DNA replication [42], [43]. Radiotherapy with ionizing radiation generates DSBs, which are later converted to ssDNA. These treatments kill the cancer cells or stop their proliferation, at least partially, by activating the DNA damage response. However, in the presence of elevated levels of FAs, the DNA damage response is compromised, which may contribute to drug resistance. Theoretically, a combinational use of these genotoxic drugs or radiotherapy with inhibitors for FASN might be beneficial, at least for pre-cancer cells when the DNA damage response pathway is still intact.

## Materials and Methods

### Isolation and culture of MEFs and calvarial osteoblasts

Mouse embryonic fibroblasts were prepared as previously described [21]. To prepare primary osteoblasts, calvaria from 19–20 day old fetuses or new born pups were isolated, washed in PBS and digested in MEM alpha medium containing collagenase type V and trypsin for 10 min at 37°C four times. The supernatant from the first digestion was discarded and supernatants from the last three digestions were pooled. The cells were washed and plated onto 6 well plates and grown in MEM alpha medium supplemented with 15% FCS (HyClone) and glutamine until confluent [21]. The osteoblast cultures were amplified to passage 3 before being used in further experiments. MEFs, HCT116, MCF-7, and NIH3T3 were cultured in DMEM supplemented with 10% FCS and penicillin/streptomycin in humidified atmosphere of 5% CO_2_ at 37°C.

### DNA damage

DNA damage was generated with genotoxic stress. 0.1–1 µM adriamycin, 10 Gy of gamma-radiation, or 5 mM hydroxyurea was used to treat different cells for different periods of time. To test the effect of SFAs or FASN inhibitors on DNA damage response, different concentrations of palmitic acid, myristic acid, stearic acid (Sigma, USA), or Cerulenin or C75 (Cayman Chemical, USA) were added to the cultures before the addition of DNA damage reagents. Briefly, for SFAs treatment, 100 µM of SFAs were included in the culture medium for overnight. The cells were then treated with adriamycin or hydroxyurea for different periods of time before being harvested for further experiments. To inhibit FASN, C75 or Cerulenin were included in the culture medium for 24 hrs. The cells were further treated with adriamycin or hydroxyurea. To knock down FASN, SiGenome ON-TARGET plus SMART pool duplex of FASN were purchased from DHARMACON (Cat# L-040091).

### Western blot analysis

Cells were washed with cold PBS and lysed in RIPA buffer containing 1 mM sodium orthovanadate, 1 mM NaF, and protease inhibitors. Protein concentration was determined by the Bio-Rad assay. For immunoblotting, proteins were resolved by SDS-PAGE and were transferred to PVDF membranes (Millipore), followed by incubation with primary and secondary antibodies and detected by ECL kit (Amersham Biosciences) [22]. The following antibodies were used in this study: β-actin (Sigma), phospho-Chk1 (Ser345), phospho-p53 (Ser15), phospho-Atr (Ser428), Chk1, FANS, and p53 (Cell Signaling), phospho-Atm (Ser1981) (Rockland), Atm and Atr (Gene Tex), H2AX (Bethyl Lab), p21 and Bax (Biochem Diagnostic).

### Cell viability analysis

To measure cell death rates, cells were plated in 96 well plates at 1×10^4^/well, and then treated with fatty acids or FASN inhibitors for 24 hrs, followed by adriamycin for different periods of time. Cell proliferation reagent WST-1 (Roche) was added to each well and the cells were further incubated for 3 hour at 37°C. The absorbance was measured against a background control by microplate (ELISA) reader at 430 nm. The reference wavelength is 650 nm.

### Immunofluorescence staining

Cells were grown overnight on glass cover-slips, subjected to adriamycin or hydroxyurea treatment, and were fixed with 4% paraformaldehyde and permeabilized with 0.1% Triton X-100 for 20 min, blocked with 5% BSA for 30 min, and then incubated with anti-H2AX antibodies and FITC-conjugated secondary antibodies. The number of foci was counted under confocal microscope.

### Statistical analysis

Statistical analysis was performed using Student's unpaired *t-*test (STATISTICA).

## Supporting Information

Figure S1 (1.96 MB DOC)

Figure S2 (1.23 MB TIF)

Figure S3 (0.67 MB TIF)

Figure S4 (1.56 MB TIF)

Figure S5 (1.44 MB TIF)

Figure S6 (0.99 MB TIF)

Figure S7 (1.00 MB TIF)

Figure S8 (0.86 MB TIF)

Figure S9 (0.45 MB TIF)
