# Supplementary figures and images for: Saturated Fatty Acids Modulate Cell Response to DNA Damage: Implication for Their Role in Tumorigenesis
Source: PLoS One. 2008 Jun 4;3(6):e2329. doi: 10.1371/journal.pone.0002329 (PMC2402972; doi:10.1371/journal.pone.0002329)

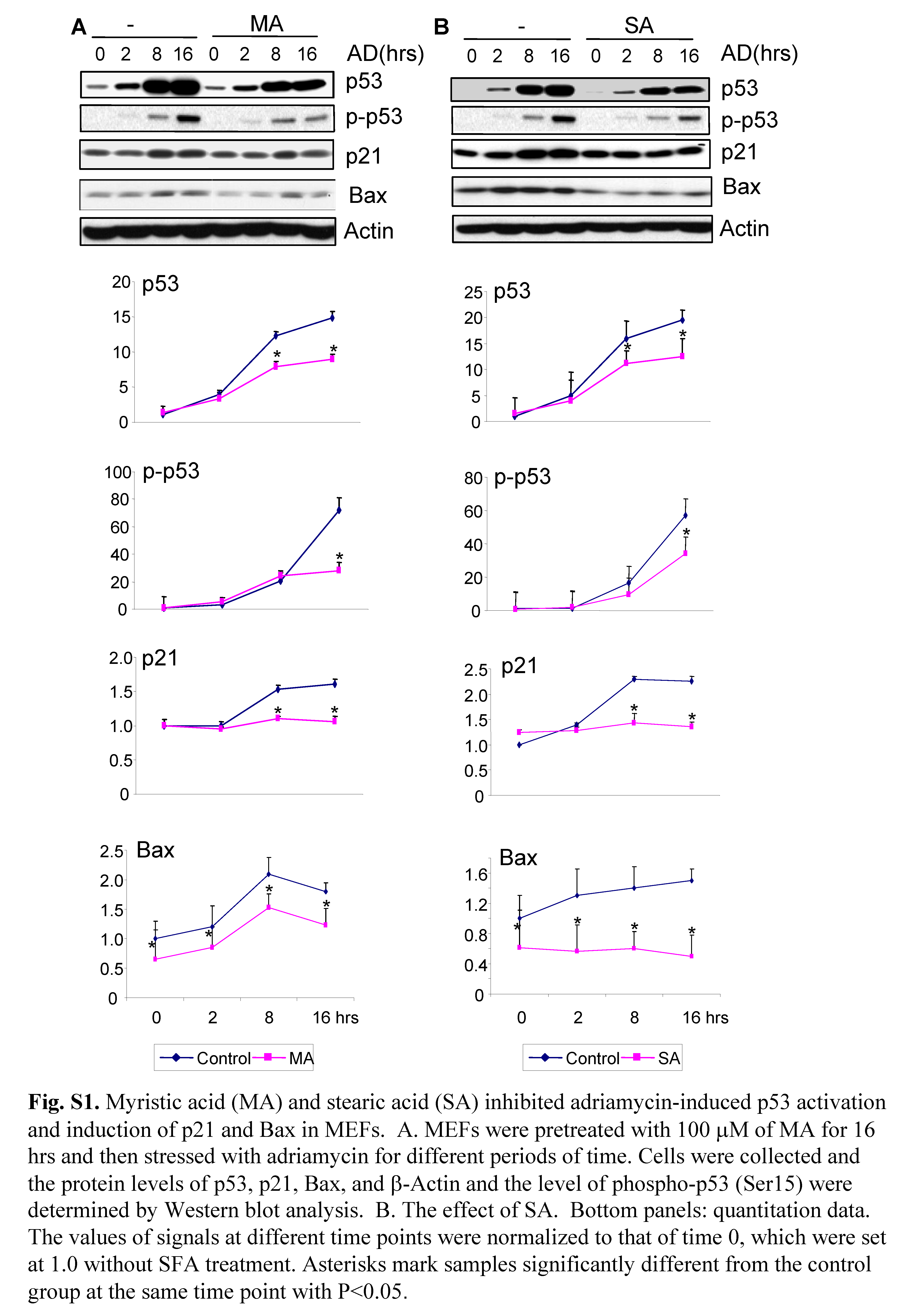

Supplement: Figure S1 — (1.96 MB DOC) [file pone.0002329.s001.tif]

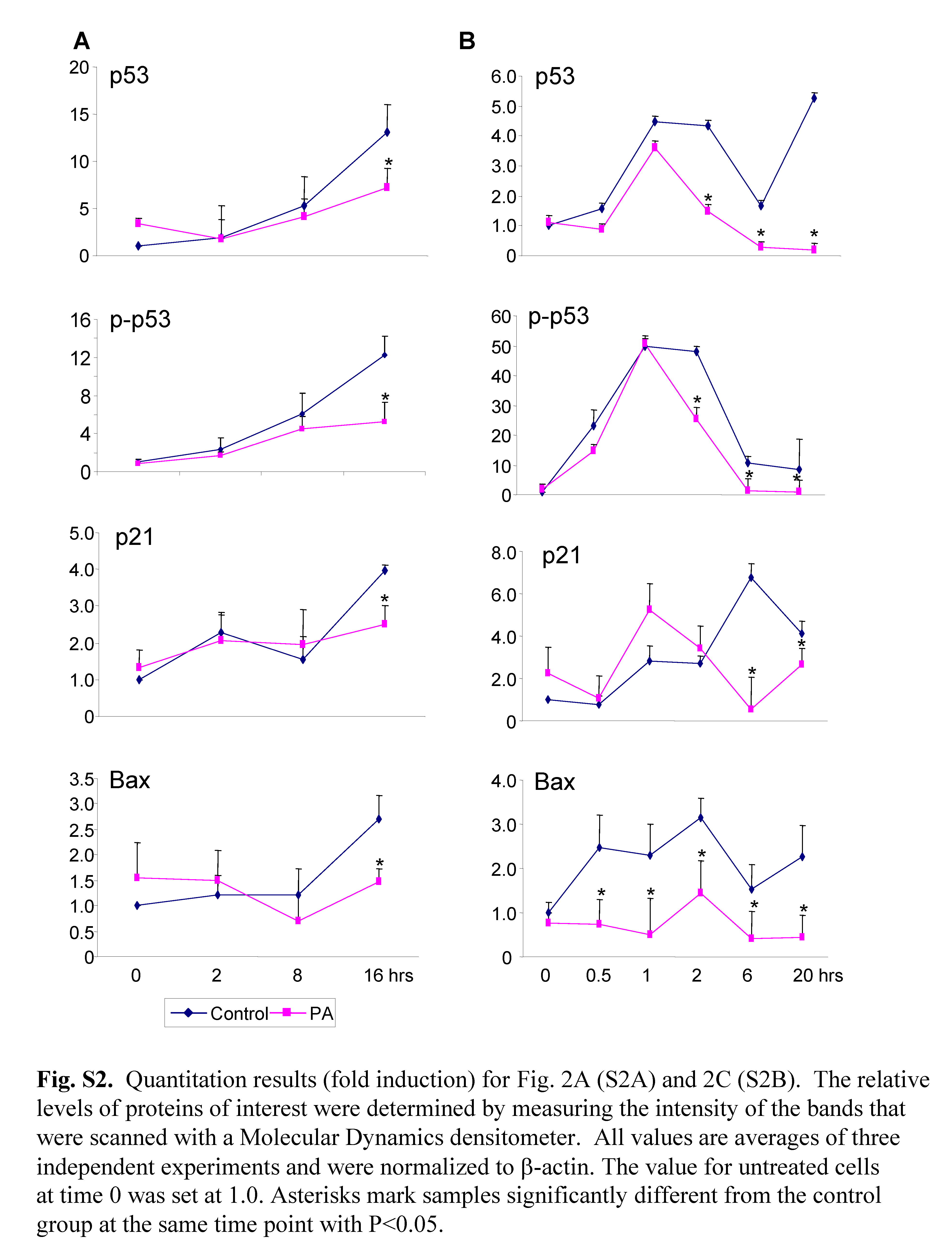

Supplement: Figure S2 — (1.23 MB TIF) [file pone.0002329.s002.tif]

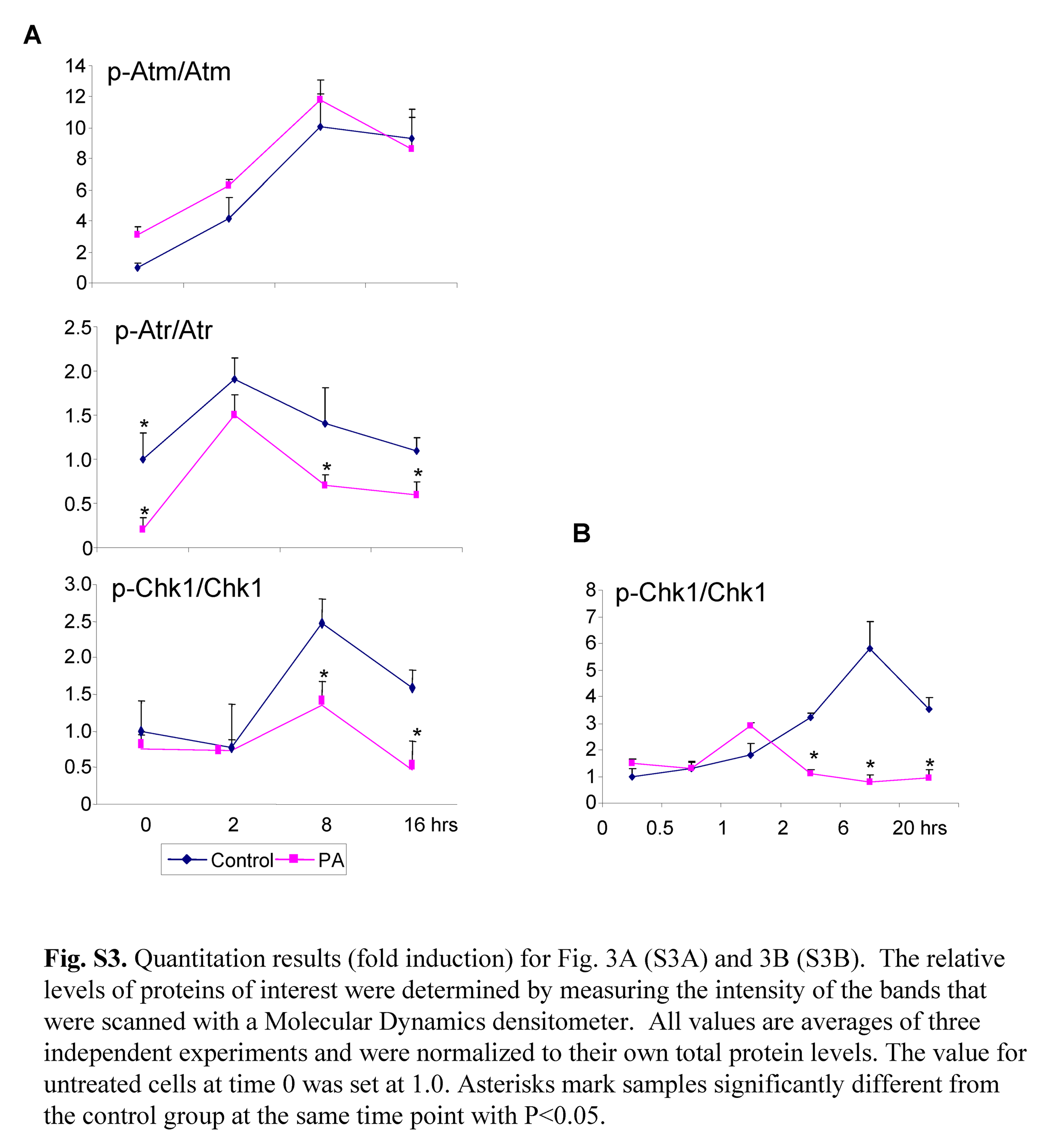

Supplement: Figure S3 — (0.67 MB TIF) [file pone.0002329.s003.tif]

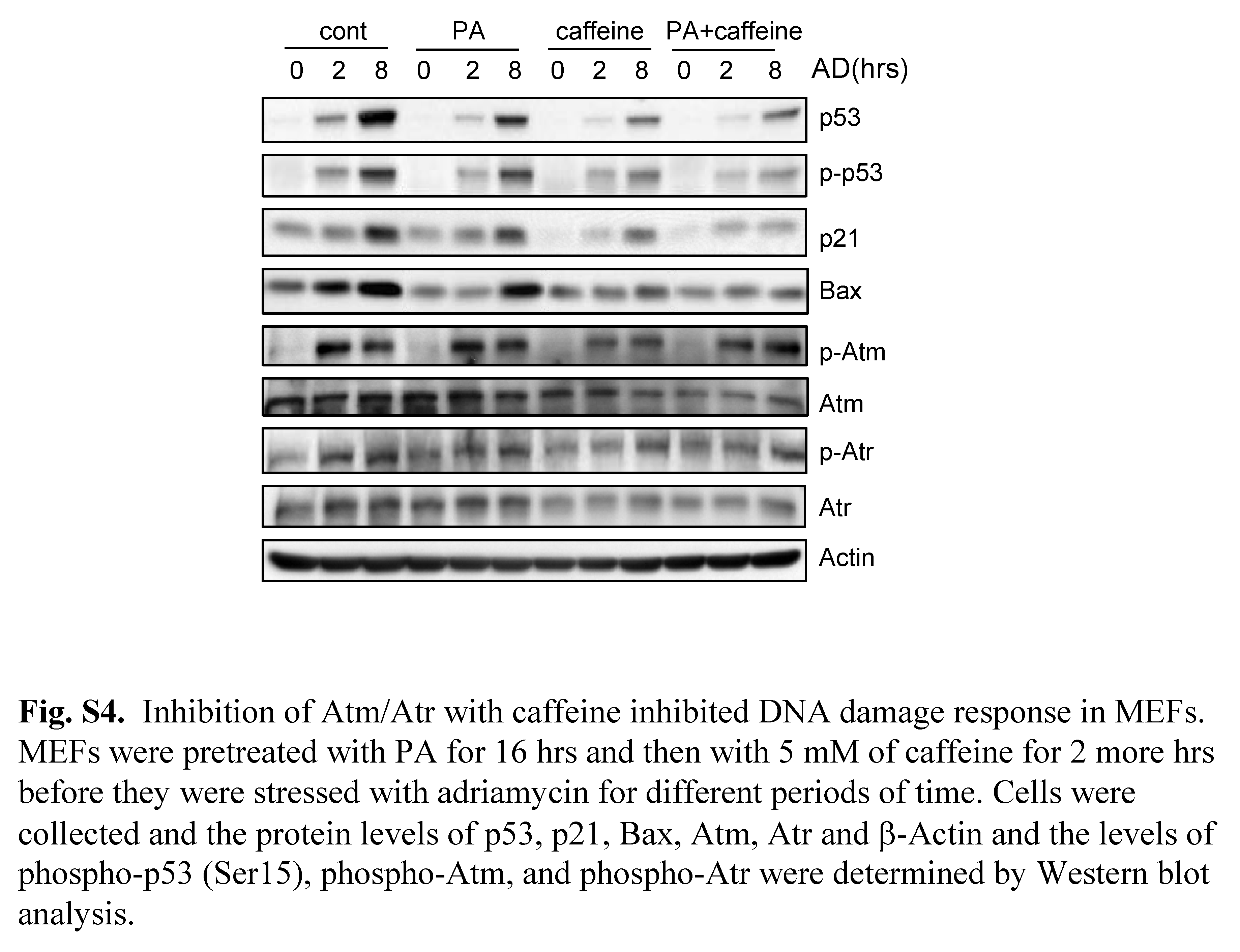

Supplement: Figure S4 — (1.56 MB TIF) [file pone.0002329.s004.tif]

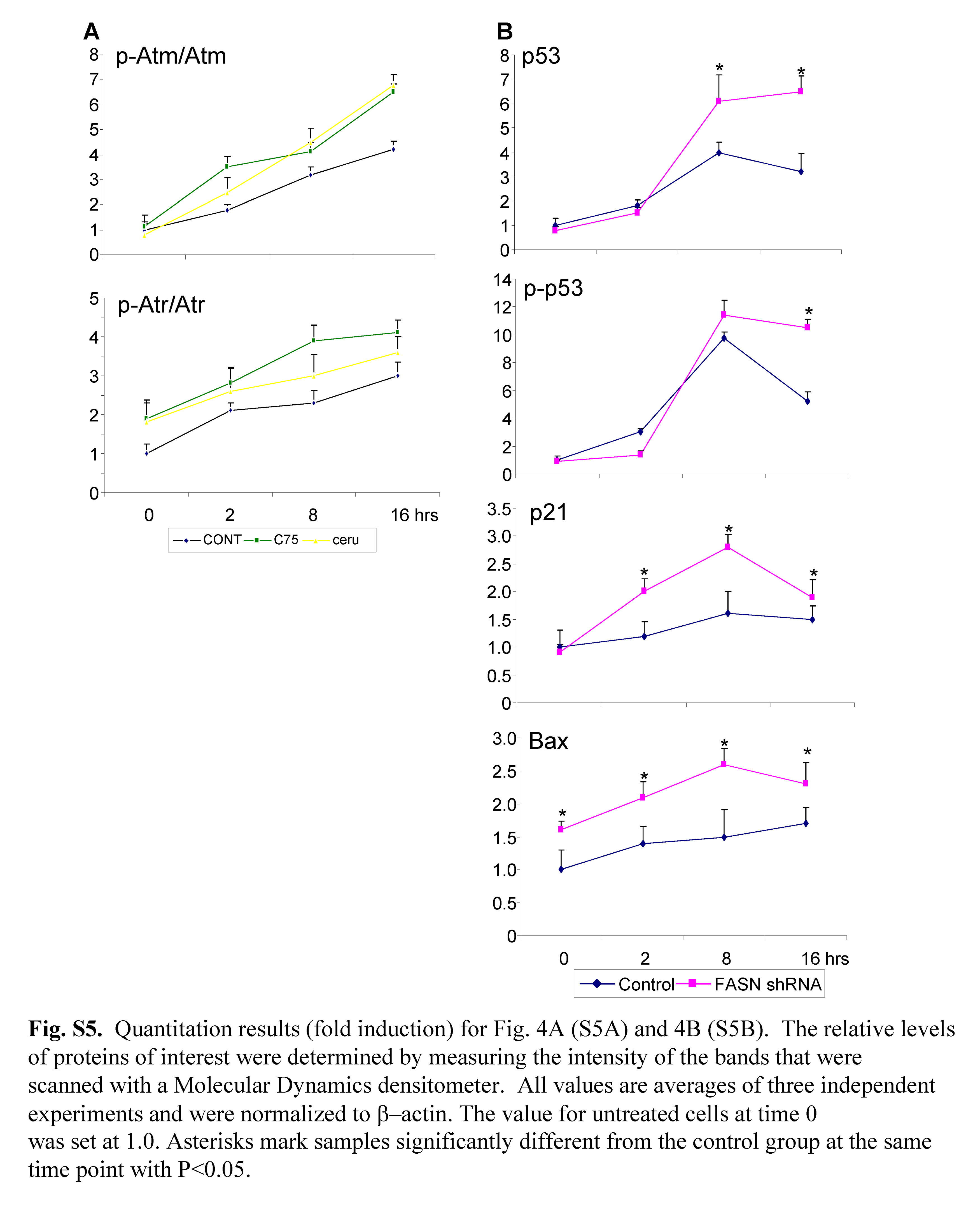

Supplement: Figure S5 — (1.44 MB TIF) [file pone.0002329.s005.png]

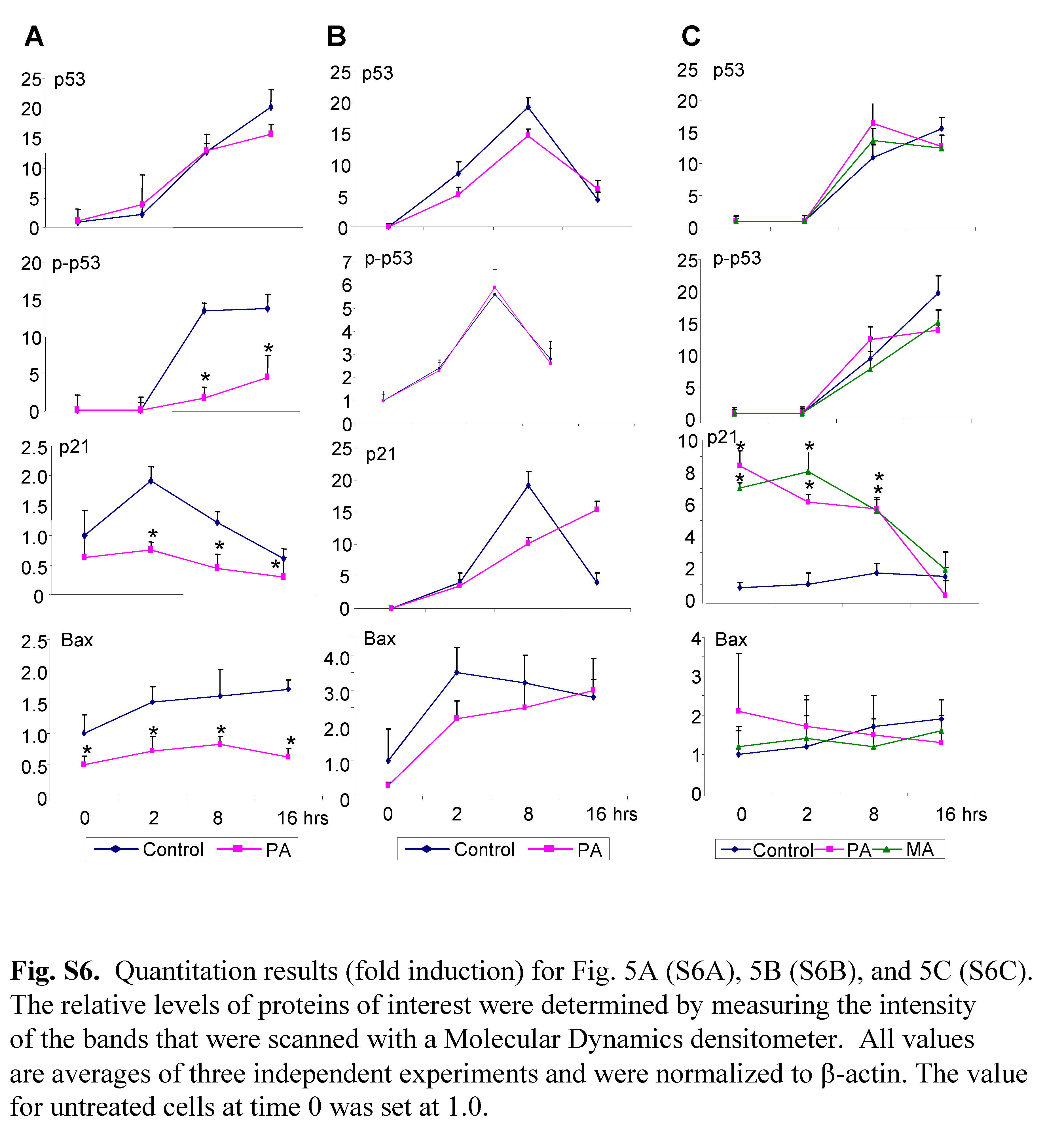

Supplement: Figure S6 — (0.99 MB TIF) [file pone.0002329.s006.tif]

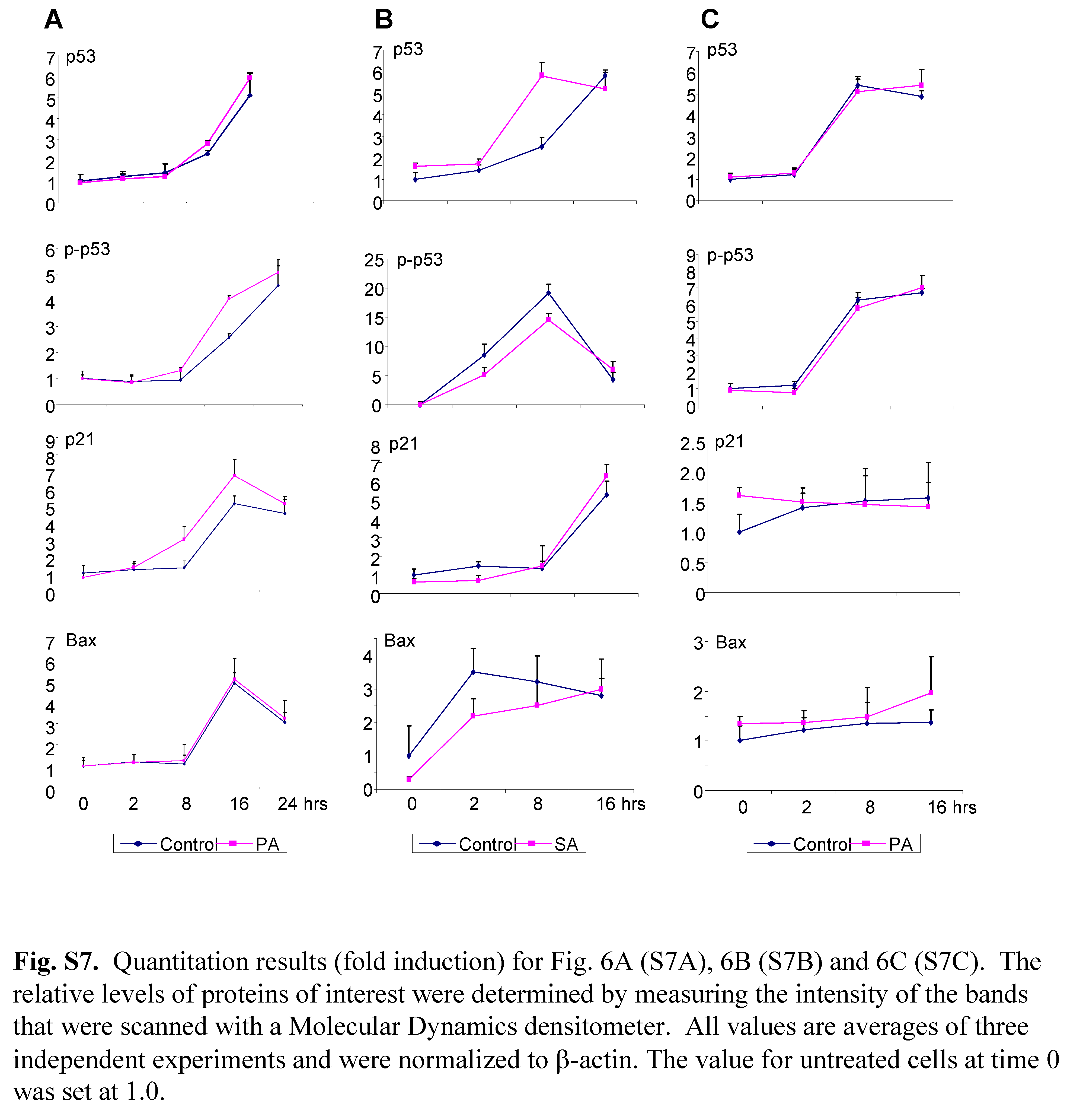

Supplement: Figure S7 — (1.00 MB TIF) [file pone.0002329.s007.tif]

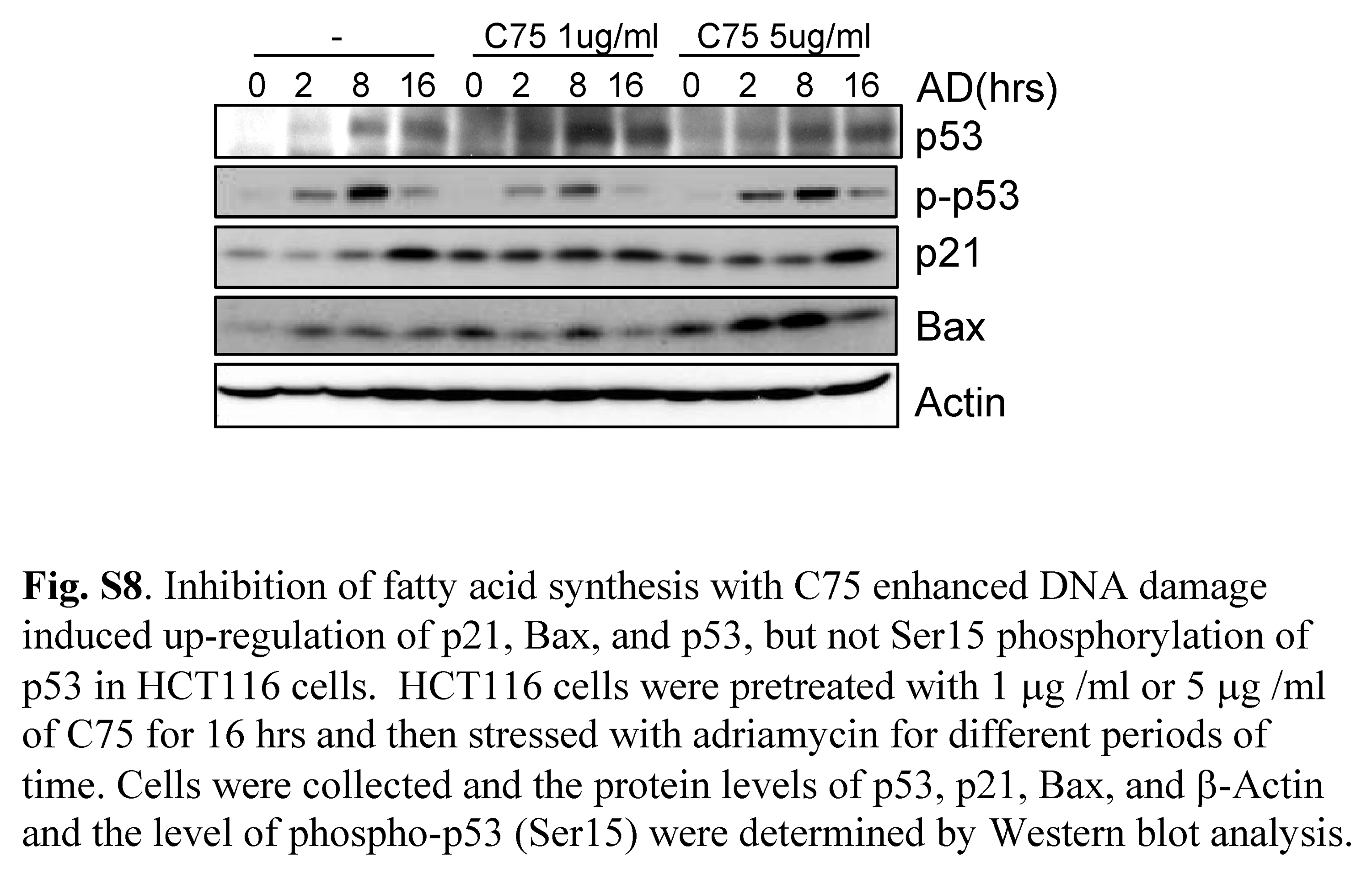

Supplement: Figure S8 — (0.86 MB TIF) [file pone.0002329.s008.tif]

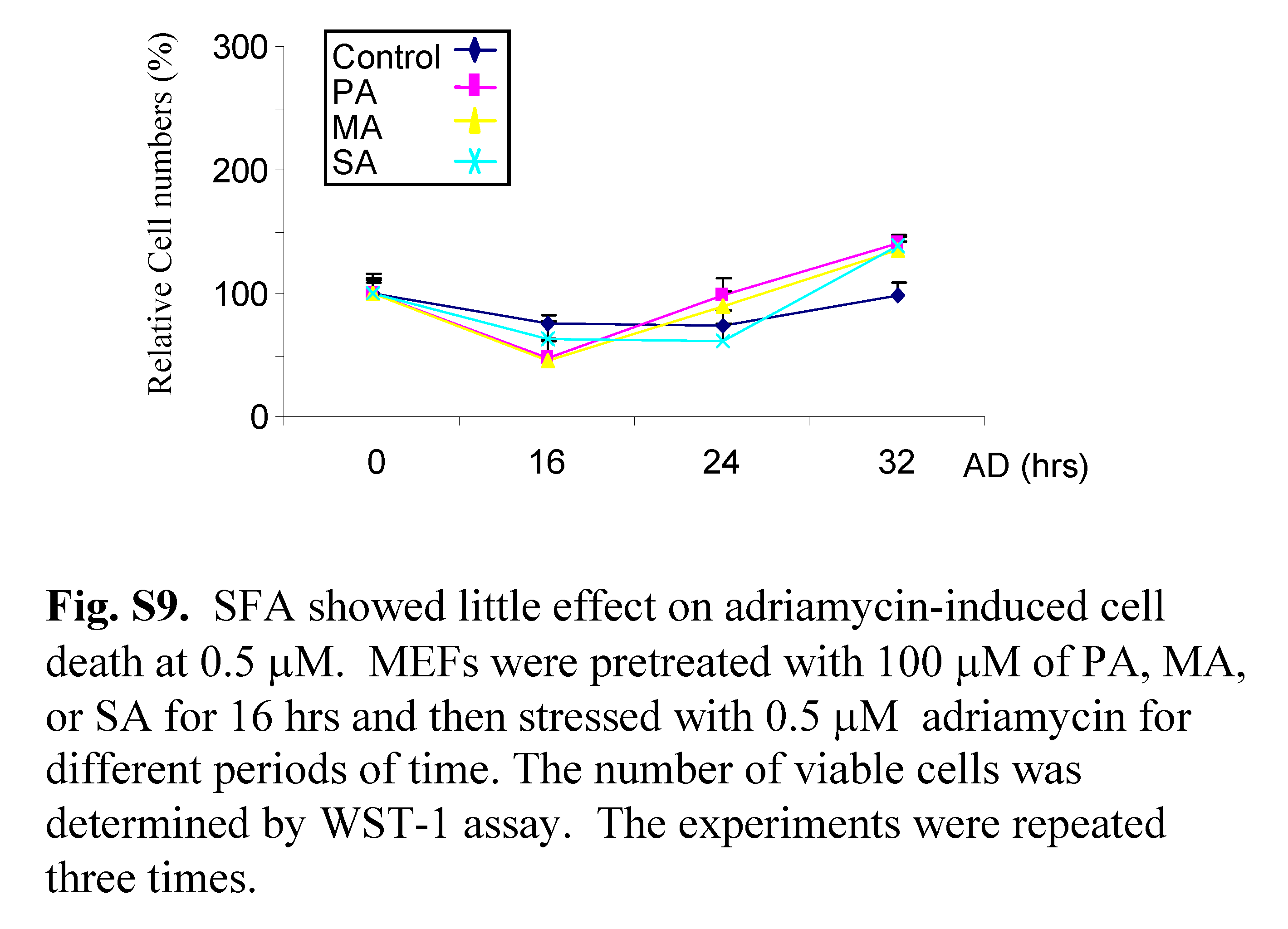

Supplement: Figure S9 — (0.45 MB TIF) [file pone.0002329.s009.tif]
